# Epidemiological Trends in the Characterization of Human Papillomavirus Virus-Related Oropharyngeal Cancer: A Global Perspective

**DOI:** 10.1007/s11912-025-01656-4

**Published:** 2025-08-20

**Authors:** Allen M. Chen

**Affiliations:** 1 Department of Radiation Oncology, Chao Family Comprehensive Cancer Center, School of Medicine, University of California, Irvine, CA 92617 USA; 2 Department of Radiation Oncology, School of Medicine, University of California, Orange, CA 92868 USA

**Keywords:** HPV, Head and neck, Cancer, Oropharyngeal, Squamous cell

## Abstract

**Purpose of Review:**

The incidence of human papillomavirus (HPV)-positive oropharyngeal cancer has increased to epidemic-like proportions in the United States and other industrialized nations. However, geographical variations are notable across the world. While significant progress has been made in the understanding of this disease with respect to its etiology, underlying biology, and pathogenesis, numerous uncertainties persist. The purpose of this review is to thus present some of the controversies and questions surrounding this disease focusing on its unique epidemiology. A selected series of critical points were presented related to the epidemiology, pathogenesis, and diagnosis of HPV-positive oropharyngeal cancer. Interpretive viewpoints were provided after a comprehensive review of the literature.

**Recent Findings:**

HPV is now estimated to cause approximately 70% of oropharyngeal cancers in the United States and other developed countries. However, in developing countries, the incidence of HPV-positive oropharyngeal cancer is significantly lower. Data has also established that HPV-positive and HPV-negative oropharyngeal cancer represents distinct entities that generally originate in different settings. Since HPV-positive oropharyngeal cancer is increasingly being recognized as a sexually transmitted disease with unique modes of transmission, the epidemiological implications are of practical relevance. The resultant geographical variation in incidence rates among countries across the world is thus believed to be due to differing lifestyles and sexual norms. Although there is currently no role for screening, efforts to promote general awareness particularly among high-risk groups should be prioritized. The development of a novel staging system specific for patients with HPV-positive oropharyngeal cancer also has important ramifications with respect to treatment.

**Summary:**

HPV-positive oropharyngeal cancer is increasingly recognized as a public health problem with a unique worldwide geographical distribution. As the etiology of this disease is increasingly elucidated, efforts to promote awareness and education are warranted.

## Introduction

The worldwide incidence of human papillomavirus (HPV)-associated oropharyngeal squamous cell carcinoma has risen dramatically in recent years reaching epidemic-like proportions in developed countries. With the increased recognition of the role that HPV infection plays in the oncogenesis of oropharyngeal cancer, significant advances have been made over the last several decades in the understanding of this disease. Most strikingly, it is now firmly established that HPV-positive oropharyngeal cancer represents a clinically and biologically distinct entity from HPV-negative oropharyngeal cancer. From a practical standpoint, the background from which HPV-positive oropharyngeal cancer emerges, as well as the risk factors for its development is uniquely different and leads to new questions regarding its prevention and diagnosis.

## Main Text

### Global Incidence

The incidence of HPV-positive oropharyngeal (throat) cancer has been shown to vary across the world, where it is more common in developed, industrialized countries and in Western societies. These discrepancies may be due to differing social norms, particularly with regard to sexual activity. In the United States, approximately 20,000 new cases are diagnosed annually, and current projections estimate a steep upward trend for the next decade with greater than 30,000 annual cases by year 2029 [1]. The overall incidence rate is approximately 5 cases per 100,000 in the United States with most new cases arising in white males aged 65 and younger, where it represents the sixth most common non-cutaneous solid cancer. Notably, the incidence of HPV-positive oropharyngeal cancer has increased dramatically over the past two decades – with studies demonstrating a three-fold increase between 2000 and 2017 [2]. Furthermore, using a national tissue repository maintained by the Surveillance, Epidemiology, and End Results program in the United States, Chaturvedi et al. showed that the percentage of all oropharyngeal cancer cases staining positive for HPV as determined by polymerase chain reaction increased dramatically from 16% during 1984 to 1989 to 72% during 2000 to 2004 [1]. In fact, oropharyngeal cancer has now eclipsed cervical cancer as the most neoplasm associated with HPV infection.

Epidemiologic data from Europe also has confirmed that the incidence of oropharyngeal cancer is on the rise in Western countries in particular. While the proportion of cases directly attributable to HPV has generally been lower than in the United States and believed to be due to higher smoking rates, the evidence is nonetheless provocative. Data from the United Kingdom has shown that between 2002 and 2011, the incidence of oropharyngeal caner increased by 101% [2]. Notably, the relative proportions of HPV-positive and negative cases remained stable over time at approximately 50% suggesting that both increased exposure of HPV in the general population as well as increasing smoking rates may underlie this observation [3]. Similarly, registry data from Denmark showed that the age-adjusted incidence rate per 100,000 increased from 1.8 in 2000 to 5.1 in 2017 with the prevalence of HPV-positive cases rising by 10% [4]. Another study from Germany showed that the proportion of newly diagnosed oropharyngeal cancer attributed to HPV has more than doubled from 2000 to 2017 [5].

In contrast, a systematic review including 3,850 patients with head and neck cancer from sub-Saharan Africa showed that only 20% of oropharyngeal cancer cases was related to HPV [6]. Another study of 266 oropharyngeal cancer patients diagnosed between 2007 and 2017 in South Africa showed that the proportion of cases harboring high-risk HPV was only 5% [7]. In another study, investigators from Nigeria reviewed the tumor specimens of 149 head and neck cancer cases and were unable to detect HPV in any of the samples [8]. Indeed, research from countries such as Mozambique and Cameroon have suggested that the relatively lower rates of HPV infection observed might reflect differing acceptances of sexual norms in those parts of the world [9, 10]. Similarly, the prevalence of HPV-positive oropharyngeal cancer in South American is also low. For instance, studies have generally reported a prevalence of between 5 and 20% for populations in Brazil [11, 12]. Data from Mexico has also suggested that the rates of HPV-positive disease are intermediate between that of Western countries and those in the developing world [13].

Epidemiological data has suggested that the prevalence of HPV-positive oropharyngeal cancer might possibly vary depending on the socioeconomic status of the country [14]. For instance, the incidence appears to be increasing in Japan, similar to Western countries [15]. Saito reported that the prevalence of HPV-positive oropharyngeal squamous cell carcinoma cases more than doubled between 2000 and 2003 and 2008–2011 from 15 to 33%, respectively [16]. Epidemiological data from China has shown similar trends [17]. For instance, findings from a population-based registry from Hong Kong showed that the proportion of oropharyngeal cancer cases related to HPV rose from 40 to 65% from 2010 to 2015 to 2016–2020 [18]. Another study from Singapore showed the prevalence of HPV-positive oropharyngeal cancer was approximately 40% [19]. In other less affluent countries such as Thailand, for instance, the rates tend to be lower. Saiya et al. reported that the proportion of oropharyngeal cancer cases that were HPV-positive between 2009 and 2020 was 15% and had stayed relatively steady over time [20]. Another study from Malaysia showed that while the prevalence of HPV-positive oropharyngeal cancer in that country was not as high as in developed nations, it was still on an upward trend [21]. Notably, in more remote countries, the incidence of HPV-positive oropharyngeal cancer has stayed low. For instance, one study from inner Mongolia showed that the prevalence of HPV-positivity was only 7% [22].

## Risk Factors

While the global variation in the prevalence of HPV-positive oropharyngeal cancer seems to be driven by socioeconomic factors and those related to sexual practices, it must be recognized that differences within the United States also exist based on demographics. For instance, the incidence of HPV-positive oropharyngeal cancer differs not only by sex but also by age and race. For instance, a higher prevalence of this disease has consistently been observed in white men than in any other subgroup. Furthermore, patients with HPV-positive oropharyngeal cancer have been shown to be younger and healthier compared to HPV-negative patients [23]. While smokers can definitely get HPV-positive oropharyngeal cancer, many patients with this disease have never smoked. In parallel with the increased incidence in white men in the United States, higher socioeconomic status has also been associated with an increased incidence [24]. For instance, one population-based registry study showed that higher levels of educational attainment and higher income were both independently associated with an increased risk of developing HPV-positive oropharyngeal cancer [25]. In short, an epidemic appears to have developed affecting younger middle-aged men of professional, educated classes. In contrast to patients with HPV-negative head and neck cancer, which has always been strongly linked with the traditional risk factors of tobacco and alcohol use, HPV-positive oropharyngeal cancer patients have a notably distinct risk profile that generally reflects sexual behavior that is considered high-risk. It is thus believed that fundamental differences between how cancer arises in the setting of virally mediated versus smoking mediated disease underlies the differing response patterns to treatment associated with each. Table 1 outlines some of the general differences between HPV-positive and HPV-negative oropharyngeal cancer with respect to patient characteristics.

**Table 1:** Generalized profiles for oropharyngeal cancer patients based on HPV status

	HPV-negative	HPV-positive
Age	Older (> 60 years)	Younger/middle-age
Risk-factors	Tobacco/Alcohol	Oral sex
Etiology	Mutation-driven	Virus-driven
P53 gene	Altered	Wild-type
Male: female ratio	1–2:1	5:1
Ethnic predilection	All	White/Caucasian
Co-morbidities	Common	Rare
Education level	Low	High
Incidence	Decreasing	Increasing

The difference between the pathogenesis of HPV-positive and HPV-negative oropharyngeal cancer is due to the fact that the former is a sexually transmitted disease. Oral HPV is transmitted to the mouth by oral sex, or possibly in other ways. While many people are exposed to oral HPV in their life, only a small minority of these will develop cancer. Population-based data has shown that approximately 10% of men and 4% of women carry the oral HPV virus asymptomatically at any given time [26]. Although nearly all people clear the virus within 1 to 2 years, HPV infection can persist in some placing them at higher risk for cancer [27]. Although it is generally believed that HPV-related oropharyngeal cancer is strongly associated with oral sex and the number of oral sex partners, investigators recently showed that a higher number of casual sex partners, younger age at first oral sex, and extramarital sex also significantly raised the risk of HPV-associated oropharyngeal cancer [28]. Additionally, these same researchers described a new metric of quantifying risk exposure as a measure of sexual intensity (sex-years) based on the number of oral sex partners per 10 years across different ages. Their data conclusively demonstrated a strong correlation between increasing sex-years with the development of HPV-positive oropharyngeal cancer, even after adjusting for cumulative number of oral sex partners. For subjects with greater than 5 sex-years, for instance, the odds of developing HPV-positive oropharyngeal cancer were nearly 3 times that of those with less than 5 sex-years exposure. How the differences in sexual norms across the world contributes to the variation in prevalence of HPV-positive oropharyngeal cancer remains an area of active investigation but has significant public health implications [29].

## Future Research

The role of HPV vaccination, while generally accepted to confer a benefit with respect to decreasing the incidence of cervical cancer in women and genital warts in men, is still uncertain in the setting of oropharyngeal cancer. In the United States, the Centers for Disease Control and Prevention currently recommends routine vaccination against HPV of boys and girls at the age of 11–12 years [30]. For children who receive their first dose before the age of 15, only two doses are necessary. Teens and young adults who start the series later, at ages 15 through 26 years, need three doses of HPV vaccine. Notably, HPV vaccination is not recommended for anybody older than age 26 years because the likelihood of being exposed to HPV is extremely high. Vaccination is generally protective against all HPV-associated cancers including possibly those of the oropharynx. Notably, vaccination can prevent cancer, but it cannot treat. In other words, it does not work against established HPV infections or against cells that have been transformed by HPV and are on their way to forming tumors.

Although research is ongoing regarding the potential utility of screening for oropharyngeal cancer in high-risk populations, there is currently no evidence demonstrating a benefit. Notably, screening is complicated by the lack of a reliable pre-invasive state as well as a paucity of robust techniques. While HPV-DNA as well as antibodies to HPV can be detected in bodily fluids including the blood and saliva of patients with HPV-positive oropharyngeal cancer, there is currently no established role for the screening of asymptomatic patients for the purpose of early detection [31, 32]. This is due to the lack of any studies demonstrating a benefit to screening. As such, questions regarding the sensitivity and specificity of any proposed methods will need to be fully vetted before the implementation of any screening modality. Unlike the case in cervical cancer in which a routine Pap smear can alert patients to the presence of pathological characteristics including HPV which could place them at higher risk for the development of cancer, no such assay exists in the setting of oropharyngeal cancer. Notably, the HPV subtypes typically associated with the development of oropharyngeal cancer (i.e. HPV-16 and HPV-18) are the same as the case for cervical cancer which suggests that analogies could be drawn with respect to the natural history of the oncogenesis of the diseases [33]. Notably, targeted screening might be particularly beneficial for populations in which resources are lacking. For instance, patients from developing countries may present with more severe symptoms due to the relative scarcity of health services as well as constraints related to education and awareness compared to those residing in developed parts of the world. In these situations, in which the tumor is diagnosed at more advanced stages, the treatment options are generally deemed to be more intensive [34]. Additionally, treatment can be complicated by logistical barriers as well as social determinants related to income, transportation, education, housing, and nutrition, among others. Given the rigorous nature of treatment for head and neck cancer, the importance of a robust support network has also been shown [35].

While evidence has now firmly established that HPV-positive oropharyngeal cancer is a unique entity with distinct clinical and molecular characteristics compared to HPV-negative oropharyngeal cancer, treatment is still variable [36]. However, data has accumulated demonstrating that patients with HPV-positive oropharyngeal cancer have a significantly improved prognosis as a result of their exquisite sensitivity to radiation compared to their HPV-negative counterparts [37]. From a practical standpoint, these tumors have been shown to shrink briskly and robustly in both the laboratory and in actual patients. Moreover, given the strong link between HPV and radiation response, the American Joint Committee on Cancer (AJCC) created a new staging system in 2016 specifically for patients diagnosed with HPV-positive oropharyngeal cancer (through p16, a widely accepted surrogate for HPV) to reflect its favorable prognosis compared to those with HPV-negative disease [38]. Notably, many HPV-positive tumors that had been previously categorized as stage IV in the prior AJCC system were significantly “down-staged” to stage II or even stage I cancers. Sharma et al. analyzed 621 patients using criteria from the most recent (eighth) AJCC staging system in comparison to that of the prior (seventh) version. Using the seventh edition, only 8% of patients were classified as having stage I or II disease. In contrast, with the most recent system, this percentage rose to 63% [39]. This new staging system has prompted many investigators to question historical treatment paradigms, which were developed and based on information that is now considered obsolete. Indeed, given that a higher proportion of patients are being diagnosed with earlier stage disease simply with the transition to a more updated classification system, the implications with respect to treatment might be significant. For instance, current options include surgery, radiation, chemoradiation, or a combined approach using more than one modality. Moreover, studies have shown that the efficacy of treatment is diluted in HPV-positive oropharyngeal cancer patients with a smoking history, which might influence decision-making in certain situations [40]. This is particularly true given the differences in tobacco consumption across the world [41]. Thus, due to the variability of treatment patterns across the globe, efforts of promoting standardized, evidence-based approaches are clearly warranted [42].

## Conclusion

The recognition that HPV-positive oropharyngeal cancer represents a uniquely different disease has ushered in a new era in the evaluation and management of patients with this diagnosis. Given its potential for patient counseling and treatment decision-making, staining for HPV is now standardly conducted on all oropharyngeal cancer specimens across the world. However, dramatic variations in the epidemiology of this disease exist with respect to prevalence, risk factors, and treatment. Based on the emerging evidence, as well as on the explosion in interest from patients and physicians alike, well-designed clinical trials with associated translational studies are urgently needed to better understand the natural history of this disease in relation to its different pattens of origination. Given the increasing incidence of this disease across a large part of the globe, the importance of this research cannot be overstated.

## Key References

- 1. Chaturvedi AK, Engels EA, Pfeiffer RM, et al. Human papillomavirus and rising oropharyngeal cancer incidence in the United States. J Clin Oncol 2023; 41: 3081–3088.
-
  **This is an important epidemiologic study demonstrating increasing rates of HPV-positive oropharyngeal cancer in the United States.
- 14. Lu Y, Xie Z, Luo G, et al. Global burden of oropharyngeal cancer attributable to human papillomavirus by anatomical subsite and geographic region. Cancer Epidemiol 2022; 78: 103,140.
-
  *Assessment of HPV-positive oropharyngeal cancer impact across the world.
- 23. Hess CB, Rash DL, Daly ME, et al. Competing causes of death and medical comorbidities among patients with human papillomavirus-positive vs. human papillomavirus-negative oropharyngeal carcinoma and impact on adherence to radiotherapy. JAMA Otolaryngol Head Neck Surg 2014; 140: 312–316.
-
  *An analysis of causes of mortality in patients with HPV-positive oropharyngeal cancer.
- 26. Gillison ML, Broutian T, Pickard RK, et al. Prevalence of oral HPV infection in the United States, 2009–2010. JAMA 2012; 307; 693–703.
-
  **Prospective study evaluating the prevalence of oral HPV infection in the general population.
- 27. Kreimer AR, Campbell CM, Lin HY, et al. Incidence and clearance of oral human papillomavirus infection in men: the HIM cohort study. Lancet 2013; 382: 877–887. **Prospective study evaluating the prevalence of oral HPV infection in the general population.
-
  **Prospective study evaluating the prevalence of oral HPV infection in the general population.

## Data Availability

No datasets were generated or analysed during the current study.

## Declarations

### Transparency Statement

The lead author (AC) affirms that this manuscript is an honest, accurate, and transparent account of the study being reported; that no important aspects of the study have been omitted; and that any discrepancies from the study as planned (and, if relevant, registered) have been explained.
